# AI-supported non-invasive measurement for intraoperative hypotension reduction in major Orthopedic and trauma surgery—study protocol for a randomized clinical trial

**DOI:** 10.1186/s13063-025-09194-x

**Published:** 2025-10-30

**Authors:** Marit Habicher, Rosa Kleymann, Karim Shakkour, Nuria Holstein, Christian Koch, Melanie Markmann, Emmanuel Schneck, Michael Sander

**Affiliations:** Department of Anesthesiology, Intensive Care Medicine and Pain Therapy, Justus Liebig University of Giessen, University Hospital of Giessen & Marburg, Rudolf-Buchheim-Street 7, Giessen, 35392 Germany

**Keywords:** Intraoperative hypotension, ClearSight HPI system, Non-invasive monitoring, AI, Major orthopedic surgery, Trauma surgery, Acute kidney injury, Myocardial injury

## Abstract

**Background:**

Intraoperative hypotension (IOH) is a common complication in non-cardiac surgeries, with significant impacts on postoperative outcomes such as acute kidney injury (AKI), myocardial injury after non-cardiac surgery (MINS), and postoperative cognitive dysfunction (delirium). Traditional non-invasive blood pressure monitoring often results in blind gaps through the discontinuous measurement, potentially missing critical hypotensive events. This study investigates the efficacy of a non-invasive, continuous AI-supported technology using the Hypotension Prediction Index (HPI) system, designed to predict and mitigate IOH, in reducing the incidence and severity of IOH and related complications in major orthopedic and trauma surgeries.

**Methods/design:**

This monocentric, randomized, prospective interventional trial will be conducted at the University Hospital Giessen. The study will include patients aged 45 years and older undergoing major orthopedic or trauma surgeries. Participants will be randomized into two groups: an intervention group receiving hemodynamic management using the ClearSight system in combination with a HPI-based treatment algorithm and a control group receiving standard care. The patients from the standard group also receive continuous cardiac output monitoring; however, this will be blinded to the anesthesiologist, to ensure the comparability of the measured hemodynamic parameters. The primary endpoint is the incidence, duration and severity of IOH, which is defined as MAP < 65 mmHg for at least one minute and which is calculated as the area under MAP < 65 mmHg and the time-weighted average (TWA) of MAP < 65 mmHg. Secondary endpoints include the occurrence of postoperative AKI (measured by KDIGO criteria), MINS (measured by high-sensitivity troponin I assays), and postoperative delirium. The study aims to recruit 150 patients, accounting for potential dropouts, to provide sufficient power to detect differences in the primary and secondary endpoints.

**Conclusion:**

This study aims to demonstrate improved intraoperative hemodynamic stability, with a reduction of hypotension and a reduced incidence of postoperative complications, potentially setting a new standard for non-invasive continuous monitoring in orthopedic and trauma patients.

**Trial registration:**

ClinicalTrials.gov NCT06291714. Registered on 4 March 2024.

**Supplementary Information:**

The online version contains supplementary material available at 10.1186/s13063-025-09194-x.

## Introduction

### Background

Blood pressure monitoring is a critical component of perioperative hemodynamic assessment. On the other hand, hypotension is not clearly defined as shown in previous studies [1]. Some studies defined hypotension as a systolic arterial blood pressure (SAP) below 100 mmHg or a mean arterial pressure (MAP) below 60 mmHg [1, 2]. Intraoperative hypotension (IOH) frequently occurs during non-cardiac surgeries and is associated with an increased risk of postoperative complications such as acute kidney injury (AKI) and myocardial injury after non-cardiac surgery [3]. Therefore, and as described above, the occurrence of hypotension displays a common intraoperative complication. The reasons can be found in vasodilatation, reduced inotropy (e.g., drug induced), bradycardia, or intravascular volume deficit (based on preoperative fasting and intraoperative blood loss). Main factors causing IOH are age, preexisting diseases (e.g., ASA > 3), duration of surgery, acuteness of surgery (e.g., emergency), anti-hypertensive medication, and anesthesia combining general, regional anesthesia, and preoperative baseline blood pressure [4, 5].

Complications related to IOH can be detected in most organ systems of which renal failure poses a relevant complication in the perioperative phase. AKI affects up to 25% of patients attending the intensive care unit (ICU) [6]. Liu et al. showed that even an episode of relative IOH is commonly associated with the occurrence of a postsurgical AKI (a decrease in SBP relative to preoperative value displayed a significant independent predictor of the development of AKI and of RIFLE classes I and II; odds ratio 1.084 for every − 1 mmHg change in SAP) [7]. As they state, “normotensive renal failure” is not common and is a rare phenomenon in the absence of septic and other complications. Traditional methods of blood pressure monitoring rely on intermittent measurements, which can miss critical hypotensive episodes due to "blind" intervals where no data is collected [8]. These intermittent measurements often fail to provide the necessary information for immediate intervention, leading to a higher incidence of IOH and its adverse consequences.

The failure to detect IOH using traditional methods is a significant problem, as IOH is correlated with substantial postoperative complications. IOH is particularly dangerous in orthopedic and trauma surgeries, where stable hemodynamic conditions are crucial for the success of the operation and the patient’s recovery. Studies have shown that even short periods of IOH can result in serious postoperative complications. For instance, Salmasi et al. demonstrated that IOH (defined as mean arterial pressure < 65 mmHg) is associated with higher rates of AKI and myocardial injury, underscoring the need for improved intraoperative blood pressure monitoring [9].

The ClearSight Hypotension Prediction Index (HPI) system is a non-invasive continuous hemodynamic monitoring (Edwards Lifesciences). This AI-supported system is capable of predicting hypotensive events, with a median lead time of approximately 2–3 min in clinical practice, although under certain conditions predictions of up to 15 min in advance have been reported. This capability allows anesthesiologists to take preventive measures before IOH occurs, thereby reducing the risk of complications. The predictive ability of the HPI system has been validated in previous studies [10], and earlier trials demonstrated that its use was associated with a significant reduction in intraoperative hypotension [11–13]. By enabling proactive management of hemodynamic stability, the ClearSight HPI system has the potential to significantly improve patient outcomes in major orthopedic and trauma surgeries. The HPI software is commercially available.

### Study hypotheses

#### Primary hypothesis

The perioperative use of non-invasive HPI-guided goal-directed therapy (GDT) reduces the incidence, and severity of IOH in patients undergoing major trauma and orthopedic surgery.

#### Secondary hypotheses

The use of HPI-guided GDT reduces the occurrence of postoperative renal failure and postoperative myocardial injury. Furthermore, the incidence of postoperative delirium can be reduced.

### Study objectives

The primary objective is to investigate whether HPI-guided GDT can reduce the incidence, duration, and severity of IOH in comparison with no GDT. Secondary objectives include assessing the impact on postoperative renal failure, myocardial injury, and delirium.

## Methodology

### Study design

This is a randomized, prospective interventional trial comparing continuous non-invasive cardiac output monitoring (ClearSight system) to standard care. The study will take place at the university hospital Giessen and Marburg, Giessen, Germany. The study protocol has been approved by the local ethics committee (EA 70/21). This trial has been registered with the NIH, U.S. National Library of Medicine at ClinicalTrials.gov (NCT06291714) on 4 March 2024. This article adheres to the Standard Protocol Items: Recommendations for Interventional Trials (SPIRIT) statement (Additional file 1) [14].

### Study population

The study will include patients aged 45 years and older undergoing major orthopedic or trauma surgery, such as reconstructive surgery of the pelvis (e.g., stabilization of fractures), total hip or knee arthroplasty, surgery of the proximal femur (e.g., stabilization of fractures), and surgery of the spine in a supine position. A further inclusion criterion is a planned anesthesia time of > 90 min in general anesthesia. Exclusion criteria include invasive blood pressure monitoring, participation in another interventional study, pregnancy and nursing mothers, surgery without controlled mechanical ventilation, patients with ASA I or IV, preexisting arterial fibrillation and known allergy against gelatine.

### Preoperative management

Patients are recruited before surgery after checking inclusion and exclusion criteria by the dedicated study team. Informed consent is obtained at this time from a study team member. Patients will be randomized 1:1 to the two groups after achieving the patient’s informed consent. Randomization will be performed using computer-generated random numbers and will be performed by the study team. Further, the following basic characteristics are obtained:

- Age, sex, height, weight, ASA score
- Pre-existing conditions (hypertension, coronary heart disease with and without history of myocardial infarction, peripheral arterial disease, renal failure, chronic obstructive pulmonary disease, diabetes)
- Previous major surgeries
- Current prescription of medication
- Furthermore, the following laboratory results will be gained:
- Blood cell count
- Global coagulatory function (internationalized ratio, thromboplastin time, fibrinogen levels)
- Parameters of renal function (creatinine, urea,)
- Parameters of cardiac function (troponin I, creatinine kinase, myoglobin, nt brain natriuretic peptide)
- Inflammatory Parameters (C-Reactive Protein, Procalcitonin)

### Perioperative management

#### Induction and maintenance of anesthesia

All patients receive the standard hemodynamic monitoring (electrocardiogram, non-invasive blood pressure, and plethysmography). Non-invasive blood pressure will be measured every three minutes by oscillometry.

Independently of the randomized study group, induction of anesthesia will be performed with fentanyl, propofol, and cis-atracurium. Dosages will be chosen according to the patient’s age and body weight as well as pre-existing diseases according to the assessment of the attending physician. After intubation, all patients are ventilated with a tidal volume of 8 ml/kg ideal body weight and with regard to the capnography (target end-tidal CO2 of 35–40 mmHg). The control group will be managed according to our SOP with the aim of an MAP > 65 mmHg. The depth of anesthesia will be measured during the whole operation with bispectral index monitoring with the goal between 40 and 60, avoiding burst suppression.

##### Intervention group

**Fig. 1:**
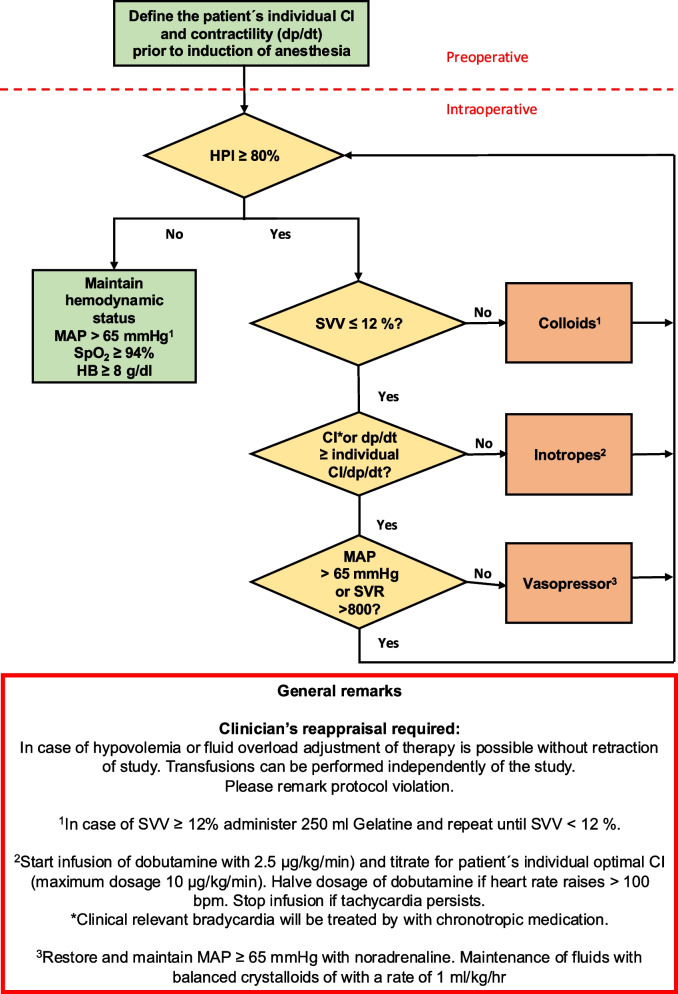
Intervention group—hemodynamic treatment algorithm

The intervention group will receive HPI-guided goal-directed therapy (GDT) using the ClearSight system. Prior to the surgery the rest cardiac index and contractility (dp/dt) will be quantified. For this purpose, the cardiac index will be measured in the preoperative night by applying the HPI ClearSight system through a study team member. If the rest cardiac index is not available throughout the night because the patient’s sleep is altered by the measurements, the awake cardiac index will be quantified until the monitoring is stopped for the night sleep of the patient. This mean baseline measurements (CI and dp/dt) will then be the target cardiac index throughout the study algorithm (Fig. 1). In case no sleep measurement is achievable, the awake measurement will be accounted as the baseline value. The perioperative study intervention period starts with the beginning of anesthesia and ends at the end of surgery. Intraoperative MAP will be maintained at least at 65 mmHg, and cardiac index and dp/dt will be individually optimized according to the GDT algorithm.

During the operation, if the HPI will increases to 80% or more, the responsible anesthesiologist will follow the algorithm (outlined below) to diagnose the underlying problem.

The algorithm includes the following steps:

1. If stroke volume variation (SVV) is ≥ 12%, a colloid bolus (250 mL) will be indicated.
2. If the cardiac index (or dp/dt) remaines below the identified individual cardiac index, inotropy therapy will be initiated with dobutamine at 2.5 µg/kg/min and titrate for patient’s individual optimal CI (maximum dosage 10 µg/kg/min). Halve dosage of dobutamine if heart rate raises > 100 bpm. Stop infusion if tachycardia persists.
3. Clinical relevant bradycardia will be treated by with chronotropic medication.
4. If the cardiac index exceeds the defined individual cardiac index, or if it is treated with dobutamine, MAP > 65 mmHg (or SVR > 800) will be restored and maintained with noradrenaline.

To ensure adherence to the algorithm in the HPI group, compliance with the study protocol will be systematically monitored. Adherence will be quantified by reviewing data from the electronic patient data management system for all hypotensive events and corresponding interventions. All hemodynamic variables will be obtained directly from the HemoSphere monitor, which is connected to every patient and records data continuously at 20-s intervals. This allows for high-resolution verification of protocol compliance and ensures that all relevant interventions in relation to hypotensive events can be reliably assessed. Specifically, all interventions triggered by the Hypotension Prediction Index (HPI) will be evaluated to ensure accurate indication and the appropriateness of the subsequent therapeutic actions. A compliance ratio will then be calculated, defined as the number of correct interventions divided by the total number of HPI-triggered therapeutic actions. This ratio will serve as a metric to assess adherence to the study protocol, specifically regarding the appropriate management of hypotensive events identified by the HPI in future practice.

##### Control group

In the control group, the HemoSphere monitor will be connected to the Clearsight finger cuff but will be fully covered, and the alarms will be silenced. The responsible anesthesiologists will treat patients based on hemodynamic variables displayed on the standard monitor. No specific protocol is employed in the control group, but the target for blood pressure monitoring is to maintain a MAP above 65 mmHg. Fluid and hemodynamic therapy is based on standard clinical practice. Colloids may be administered if the attending anesthesiologist assumes a relevant fluid deficit, for example, in cases of persistent hypotension or increasing vasopressor requirements despite crystalloid infusion. Vasopressors are typically given as bolus doses; if repeated administration becomes necessary, continuous infusion may be initiated. Inotropes are rarely used in this group, as cardiac output is not routinely measured. They may be considered only in cases of suspected impaired cardiac function (e.g., relevant pre-existing disease or intraoperative echocardiographic findings).

##### Data collection

**Fig. 2:**
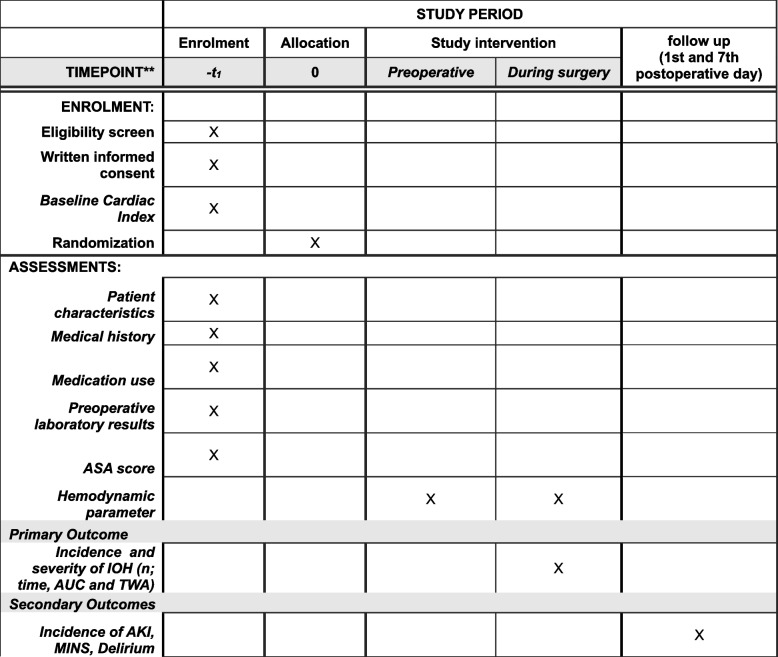
Data collection

Data will be collected by the study team preoperatively, intraoperatively, and at multiple time points postoperatively (directly postoperative at the recovery room, 1 postoperative day, 7 postoperative days, or day of discharge, whichever comes first) (Fig. 2). The data collection includes the baseline patients’ characteristics, including blood pressure measurements, renal function markers, and cardiac biomarkers as mentioned before. The intraoperative data will be collected from the HemoSphere monitor, which will collect all hemodynamic data every 20 s, and the electronic patient data management system (NarkoData Version 4.17.12.23066, IMESO, Gießen, Germany). All data will be collected in an electronic database on the university hospital server and will be only available for the study team members.

#### Outcome

**Fig. 3:**
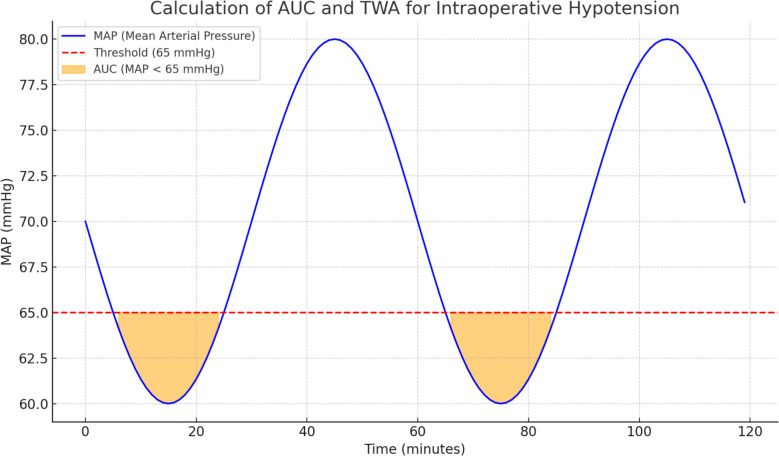
Calculation of AUC and TWA for intraoperative hypotension

The primary outcome is the incidence and severity of IOH in patients undergoing major trauma and orthopedic surgery. The severity of IOH will be quantified using the TWA of hypotension, defined as episodes where MAP remains below 65 mmHg for at least one minute. The TWA will be calculated as the area under the curve (AUC) for MAP values below 65 mmHg, normalized by the total duration of anesthesia. The AUC for MAP below 65 mmHg will be determined by multiplying the magnitude of deviation (in mmHg) below 65 by the duration (in minutes) of each hypotensive episode (see Fig. 3).

Secondary outcome parameter are:

1. Composite endpoint: Occurrence of AKI according to KDIGO criteria during the hospital stay or until postoperative day 7
  and MINS: Defined as increased troponin levels that exceed the 99th percentile of the upper reference limit of the assay after measured directly postoperative, 1^st^ and 7^th^ postoperative day or day of discharge, whatever comes first.
2. Incidence of delirium measured directly postoperative and on the 1^st^ postoperative day by the 3D-CAM Test [15]

Furthermore, intraoperative data, such as anesthesia and surgery time, volume of intravenous fluids (crystalloids, colloids, blood products, and autologous blood), blood loss, transfusion rate, type and dosage of vasopressors, and type and dosage of inotropic medication used during the procedure, were documented.

Finally, the length of hospital and ICU stay and hospital mortality were analyzed.

In addition to the predefined parameters, we will also retrospectively analyze variables related to overtreatment. In particular, we will calculate the area above threshold (AAT), defined as the cumulative time–pressure product with a mean arterial pressure above a threshold of 100 mmHg. This parameter will allow us to assess the extent of potential overtreatment in the intervention group compared to the control group.

### Sample size calculation

The study aims to enroll 150 patients to ensure adequate power. The aim of the study is to show the impact of non-invasive cardiac output monitoring on the incidence of IOH in a cohort of trauma and orthopedic surgery. Sample size calculation was performed with regard to a study by Maheshwari et al. who investigated the effect of HPI on the prevention of hypotension [16]. This study was chosen because the primary endpoint, respectively the definition of hypotension (MAP < 65 mmHg), was identical to our study, and they investigated also non-cardiac surgical patients. In this study, the mean area under the curve of MAP ≤ 65 mmHg of patients without hemodynamic management accounted for 34.2 mmHg min (8.5–112.7) compared to 32.7 mmHg min (6.3–102) in patients with hemodynamic measurement. Aiming for an alpha of 0.05 and power of 0.95, the sample size calculation resulted in 66 patients per study group (total 132 patients, based on the use of the Wilcoxon test). In order to address potential dropouts (estimated drop-out rate 10–20%), we chose to increase the patient numbers to 75 patients in each study group. In addition, if the number of patients with secondary endpoints is sufficient, a subgroup analysis of these patients is planned.

### Statistical analysis

Next to the target parameters, data of the hemodynamic and respiratory function will be achieved as well as of the anesthetic and hemodynamic management. Furthermore, general characteristics such as age, gender, body mass index, as well as pre-existing conditions and prescriptions will be assessed.

For the target variables, the results will be investigated and analyzed descriptively (e.g., checked for distribution). Metric characteristics (mean and standard deviation). as well as median and interquartile difference, and achieved frequencies (with a percentage specification) will be determined. As part of the exploratory analysis, the structural equilibrium (homogeneity) of the treatment groups will also be checked.

The distribution of parameters will be assessed using the Shapiro test, and *T *test and Wilcoxon test will be utilized accordingly for calculating group differences, considering a two-tailed value of *p* < 0.05 to be statistically significant. Categorial variables will be tested for statistically significant differences by Fisher’s exact test.

## Discussion

The present study aims to evaluate the efficacy of the non-invasive cardiac output monitoring using HPI in reducing the incidence, duration, and severity of IOH and its associated postoperative complications, such as AKI, MINS, and delirium, in patients undergoing major orthopedic and trauma surgery.

The ClearSight system by Edwards Lifesciences The part of critical care systems of Edwards Lifesciences was bluid by BD this year, so I do not if we should give an hint to that in brackets after the company name has gained attention for its capacity to provide non-invasive, continuous hemodynamic monitoring, which is essential for preventing IOH and reducing associated postoperative complications. This technology uses a finger-cuff sensor to monitor critical parameters like cardiac output and blood pressure, without the need for invasive arterial lines, making it a safer and more comfortable option for patients. One of the key features is its integration with the HPI, which predicts hypotensive events up to 15 min before their onset. This early warning system allows for timely interventions to maintain stable hemodynamic conditions [10].

Additionally, limited randomized studies indicate that avoiding hypotension may lower the risk of postoperative organ failure [17, 18]. If the HPI software effectively reduces hypotensive events, it could potentially decrease the incidence of severe complications.

In the blinded control group, we have opted not to offer advanced hemodynamic data, such as cardiac output, because these metrics are not typically available to our clinicians when using non-invasive oscillometric blood pressure monitoring. We fully acknowledge that continuous non-invasive blood pressure monitoring in both groups would provide a fairer comparison; however, this is not yet routine standard monitoring in our clinical practice. Future studies should evaluate whether continuous non-invasive monitoring alone improves outcomes, even without the HPI algorithm.

### Comparison with previous studies

Previous research has shown promising results regarding the predictive capabilities of the HPI system. Hatib et al. demonstrated the HPI’s ability to predict IOH with a sensitivity of 88% and a specificity of 87%, highlighting its potential for proactive hemodynamic management during surgery [10]. These findings underscore the potential benefits of incorporating the HPI system into routine surgical care to enhance patient outcomes. While several trials suggest that HPI-guided management can reduce the depth and duration of intraoperative hypotension, we acknowledge the ongoing debate regarding the underlying prediction algorithm. A recent position paper argued that aspects of the original validation may have introduced selection bias and called for re-validation or redevelopment of the algorithm—potentially offering no added value over simple MAP-based alerts in some scenarios [19]. Complementing this critique, a large re-analysis comparing “backward” with clinically oriented “forward” validation found that although overall AUCs remained high [20].

Furthermore in a study by Maheshwari et al., the use of the ClearSight system in high-risk non-cardiac surgery patients resulted in a significant reduction in the duration of IOH. The study found that continuous non-invasive blood pressure monitoring allowed earlier interventions in comparison with non-invasive oscillometric measurements, thereby minimizing the extent of hypotensive episodes [21]. This is particularly relevant for major orthopedic and trauma surgeries where maintaining stable hemodynamics is crucial.

In the DETECT randomized controlled trial, Kouz et al. assessed, besides the accuracy of the ClearSight system compared to intermittent oscillometric arterial pressure monitoring, that this system enabled earlier detection of IOH during the induction of anesthesia and surgery, thereby significantly reducing the duration and severity of hypotensive episodes. Importantly, patients monitored with ClearSight had fewer IOH events, suggesting that continuous monitoring offered by this system enhances intraoperative hemodynamic stability. So, it can be assumed that if the blood pressure is measured discontinuously and non-invasively the anesthesiologist will miss some relevant hypotensive episodes during the induction of anesthesia [8].

### Impact on postoperative complications

The incidence of postoperative AKI and MINS is a significant concern in major surgeries. Previous studies have linked IOH to an increased risk of these complications. For example, a study by Salmasi et al. demonstrated that even brief periods of hypotension (MAP < 65 mmHg) during surgery were associated with higher rates of postoperative AKI and myocardial injury [9].

By enabling timely interventions through predictive monitoring, using an early warning system, such as the HPI technology, has the potential to mitigate these risks.

The ClearSight system's continuous monitoring capabilities could lead to better perioperative management strategies. However, most studies using ClearSight focus on comparing its accuracy to invasive methods rather than directly examining outcomes like the reduction of hypotensive events or the need for vasopressor support in large surgical populations.

One relevant study looked at the use of the noninvasive system combined with the HPI in gynecologic oncologic surgery. This study demonstrated the ability to provide continuous blood pressure measurements and the potential for timely interventions based on hypotension prediction. However, the study primarily focused on the accuracy of the ClearSight system’s predictions and its ability to detect impending hypotensive events. It demonstrated a sensitivity of 88% and a specificity of 87% in predicting such events [22].

This suggests that the HPI-guided hemodynamic management can lead to more stable blood pressure control, reducing the likelihood of adverse postoperative outcomes.

The findings from this study could have significant implications for clinical practice. If the ClearSight HPI system proves effective in reducing IOH and its complications, it may become a standard tool for intraoperative monitoring in major orthopedic and trauma surgeries. This could lead to widespread adoption of AI-supported non-invasive monitoring technologies, enhancing patient safety and improving surgical outcomes.

### Limitations

The study may face several limitations that could influence the interpretation of its future results. As a monocentric trial, the findings will reflect the practices and patient populations of a single institution, potentially limiting their generalizability. A multicenter approach could provide more comprehensive insights. Additionally, while the chosen sample size of 150 patients is calculated to detect primary outcomes, it may lack the statistical power to observe rarer complications, such as reductions in AKI or MINS. The study design includes blinding advanced hemodynamic data from the control group while providing detailed monitoring for the intervention group, which may introduce biases in clinical management due to the differing availability of information. Moreover, the intervention group in our study combines continuous non-invasive monitoring with goal-directed therapy (targeting CI and SV) and the HPI early-warning alarm; therefore, observed effects should be interpreted as the result of this multimodal strategy rather than HPI alone. Furthermore, the outcomes will depend on the ClearSight system’s accuracy and functionality, meaning that any technical inconsistencies could affect the results.

The focus on short-term outcomes may overlook the impact on long-term recovery and organ function, including sustained renal and cardiac health. Finally, the variability in patient characteristics, due to the inclusion of different types of orthopedic and trauma surgeries, could add complexity when attributing results directly to the intervention.

## Conclusion

In conclusion, the integration of the ClearSight HPI system into intraoperative care has the potential to significantly improve hemodynamic stability and reduce the incidence of postoperative complications. This study aims to contribute to the growing body of evidence supporting the use of AI-driven predictive monitoring in enhancing surgical patient care.

## Trial status

Recruitment began in July 2024 and will be completed approximately at the end of 2025. This article is based on the most recent version of the study protocol 1.1 from 15.11.2023.

## Supplementary Information

Supplementary Material 1: SPIRIT checklist.

## Data Availability

All relevant data that will be analyzed during this study will be included in the final study publication. The datasets that will be used and/or analyzed during the study will be available from the corresponding author on reasonable request.

## Abbreviations

AI: Artificial intelligence
AKI: Acute kidney injury
AUC: Area under the curve
CI: Cardiac index
GDT: Goal-directed therapy
HPI: Hypotension prediction index
ICU: Intensive care unit
IOH: Intraoperative hypotension
MAP: Mean arterial pressure
MINS: Myocardial injury after non-cardiac surgery
SAP: Systolic arterial pressure
SVR: Systemic vascular resistance
SVV: Stroke volume variation
TWA: Time weighted average
